# Surviving Sepsis Campaign 2012 3-hour bundle in the emergency department: compliance and impact of pathway of care before and after implementation

**DOI:** 10.1186/cc14020

**Published:** 2014-12-03

**Authors:** J Masse, A Filali, O Nigeon, N Van Grunderbeeck, P Gosselin, L Tronchon, J Mallat, D Thevenin

**Affiliations:** 1 Emergency Department, Hospital Center of Lens, France; 2 Intensive Care Unit, Hospital Center of Lens, France; 3 Emergency Department, Lille University Hospital, Lille, France

## Introduction

Compliance with the Surviving Sepsis Campaign 2012 (SSC) bundle in the emergency department (ED) is a key point to improve outcome of severe sepsis and septic shock [1,2]. Before and after education of ED staff, we registered compliance and timing of lactate dosing, blood culture sampling, empiric antibiotic therapy (ATB) and fluid resuscitation, the 3-hour (H3) bundle. Survival and compliance according to the initial pathway of care were also studied.

## Methods

A monocentric study before and after education of ED staff about SSC bundles (courses, posters, pocket guides). We looked at compliance of the H3 bundle items in a retrospective and a prospective cohort, timing of realisation, day 28 survival, overall severity (SAPS2, SOFA and RISCC scores), impact of prehospital medical management, and initial pathway of care. Statistical analysis was performed with Fisher exact test and Mann-Whitney test. Multivariate analysis of factors associated with survival was made through logistic regression.

## Results

Eighty-nine patients were included in the prospective cohort, 65 in the retrospective cohort. Patterns of patients in the retrospective and prospective cohort were respectively: sex ratio M/F 29/39 and 39/47 (NS); median age 63.29/61.38 (NS); SAPS2 44/40 (*P *= 0.019); SOFA 4/3 (*P *= 0.005); RISCC 9/12.5 (*P *= 0.002). Compliance with the H3 bundle items before and after intervention was: lactate 72.1% vs. 81.4% (NS); blood cultures 61.8% vs. 67.4% (NS); ATB 29.3% vs. 52.3% (*P *= 0.005); fluids 52.9% vs. 59.3% (NS). Median delays before and after implementation were (in minutes): lactate 56 vs. 40 (*P *= 0.024); blood cultures 68 vs. 75 (NS); ATB 229 vs. 160 (NS); fluids 100 vs. 74 (NS). Survival was superior after intervention 67.6 vs. 81.4% (*P *= 0.049), and associated with a low SAPS2 score in multivariate analysis. Admission through a prehospital medical team was associated with a stronger H3 ATB compliance before intervention (*P *= 0.032). Within the ED, initial orientation to the acute care unit was associated with a better H3 ATB compliance compared to standard care before and after staff education (*P *= 0.001; *P *= 0.003), and with better overall compliance (*P *= 0.004; *P *= 0.026).

## Conclusion

Compliance with the SSC H3 bundle was increased but still needs to be improved. There is an impact of the initial pathway of care on compliance of the bundle, and on timing of ATB injection. Differences in healthworker/patient ratio in the units of care could explain these disparities [3]. Improvement could be obtained through optimizing early screening, correct initial guidance, or with dedicated teams.

## References

[B1] Dellinger RP Levy MM Rhodes A Annane D Gerlach H Opal SM Sevransky JE Sprung CL Douglas IS Jaeschke R Osborn TM Nunnally ME Townsend SR Reinhart K Kleinpell RM Angus DC Deutschman CS Machado FR Rubenfeld GD Webb S Beale RJ Vincent JL Moreno R Surviving Sepsis Campaign Guidelines Committee including The Pediatric Subgroup Surviving Sepsis Campaign: international guidelines for management of severe sepsis and septic shock, 2012 Intensive Care Med 2013 39 165 228 10.1007/s00134-012-2769-8 23361625 PMC7095153

[B2] van Zanten AR Brinkman S Arbous MS Abu-Hanna A Levy MM de Keizer NF Netherlands Patient Safety Agency Sepsis Expert Group Guideline bundles adherence and mortality in severe sepsis and septic shock Crit Care Med 2014 42 1890 1898 10.1097/CCM.0000000000000297 24670937

[B3] Shin TG Jo IJ Choi DJ Kang MJ Jeon K Suh GY Sim MS Lim SY Song KJ Jeong YK The adverse effect of emergency department crowding on compliance with the resuscitation bundle in the management of severe sepsis and septic shock Crit Care 2013 17 R224 10.1186/cc13047 24093643 PMC4055965

